# Bilateral Vocal Fold Paralysis in a Patient With Neurosarcoidosis: A ChatGPT-Driven Case Report Describing an Unusual Presentation

**DOI:** 10.7759/cureus.37368

**Published:** 2023-04-10

**Authors:** Christopher A Guirguis, Jason R Crossley, Sonya Malekzadeh

**Affiliations:** 1 Otolaryngology - Head and Neck Surgery, MedStar Georgetown University Hospital, Washington, D.C., USA

**Keywords:** chatgpt, inflammatory disease, granulomas, bilateral vocal fold paralysis, neurosarcoidosis, case report

## Abstract

This ChatGPT-driven case report describes a unique presentation of neurosarcoidosis. The patient, a 58-year-old female, initially presented with hoarseness and was found to have bilateral jugular foramen tumors and thoracic lymphadenopathy. Imaging revealed significant enlargement and thickening of the vagus nerve and a separate mass of the cervical sympathetic trunk. The patient was referred for an ultrasound-guided biopsy of the abnormal neck masses to establish a pathologic diagnosis. The patient subsequently underwent neck dissection for exposure of the vagus nerve and isolation of the great vessels in preparation for a transmastoid approach to the skull base. The presence of multifocal tumors prompted the need for a biopsy, which ultimately revealed sarcoid granulomas in the nervous system. The patient was diagnosed with neurosarcoidosis. This case highlights the potential for sarcoidosis to affect the nervous system, with multiple cranial nerve involvement, seizures, and cognitive impairment. It also emphasizes the need for a combination of clinical, radiological, and pathological findings for an accurate diagnosis of neurosarcoidosis. Additionally, this case highlights the utility of natural language processing (NLP), as the entire case report was written using ChatGPT. This report serves as a comparison of the quality of case reports generated by humans versus NLP algorithms. The original case report can be found in the references.

## Introduction

Neurosarcoidosis is a rare and complex disorder that affects the central and peripheral nervous systems. It is a form of sarcoidosis, a chronic inflammatory disease that can affect multiple organs in the body. The prevalence of neurosarcoidosis is unknown, but it is estimated to affect between 0.5% and 5% of patients with sarcoidosis. The exact cause of neurosarcoidosis is not well understood, but it is believed to be an immune-mediated disorder. The symptoms of neurosarcoidosis can vary widely and can include neurological symptoms such as seizures, headaches, and cognitive impairment, as well as visual disturbances and spinal cord involvement. The diagnosis of neurosarcoidosis can be challenging, as it requires a combination of clinical, radiological, and pathological findings [1].

Radiological findings in neurosarcoidosis include abnormalities in the brain and spinal cord, such as lesions, nodules, and inflammatory changes. These lesions are typically non-specific and can mimic other neurological disorders such as multiple sclerosis or brain tumors. Magnetic resonance imaging (MRI) is often used to detect these lesions and can show high signal intensity on T2-weighted images. Computed tomography (CT) scans can also be used to detect lesions in the brain and spinal cord, but they are less sensitive than MRI.

Histologic findings in neurosarcoidosis include the presence of non-caseating granulomas in the nervous system tissue. These granulomas are composed of inflammatory cells and are characteristic of sarcoidosis. However, a biopsy is not always necessary for the diagnosis of neurosarcoidosis and the diagnosis can be made based on the combination of clinical, radiological, and laboratory findings [1,2].

Bilateral vocal fold paralysis (BVFP) is a condition in which both vocal folds are unable to move normally. It is a rare disorder that can cause symptoms, including hoarseness, breathiness, and difficulty speaking. The anatomic basis of BVFP is complex and involves the interaction of several structures, including the vagus nerve and the muscles of the larynx [3].

## Case presentation

**Figure 1:**
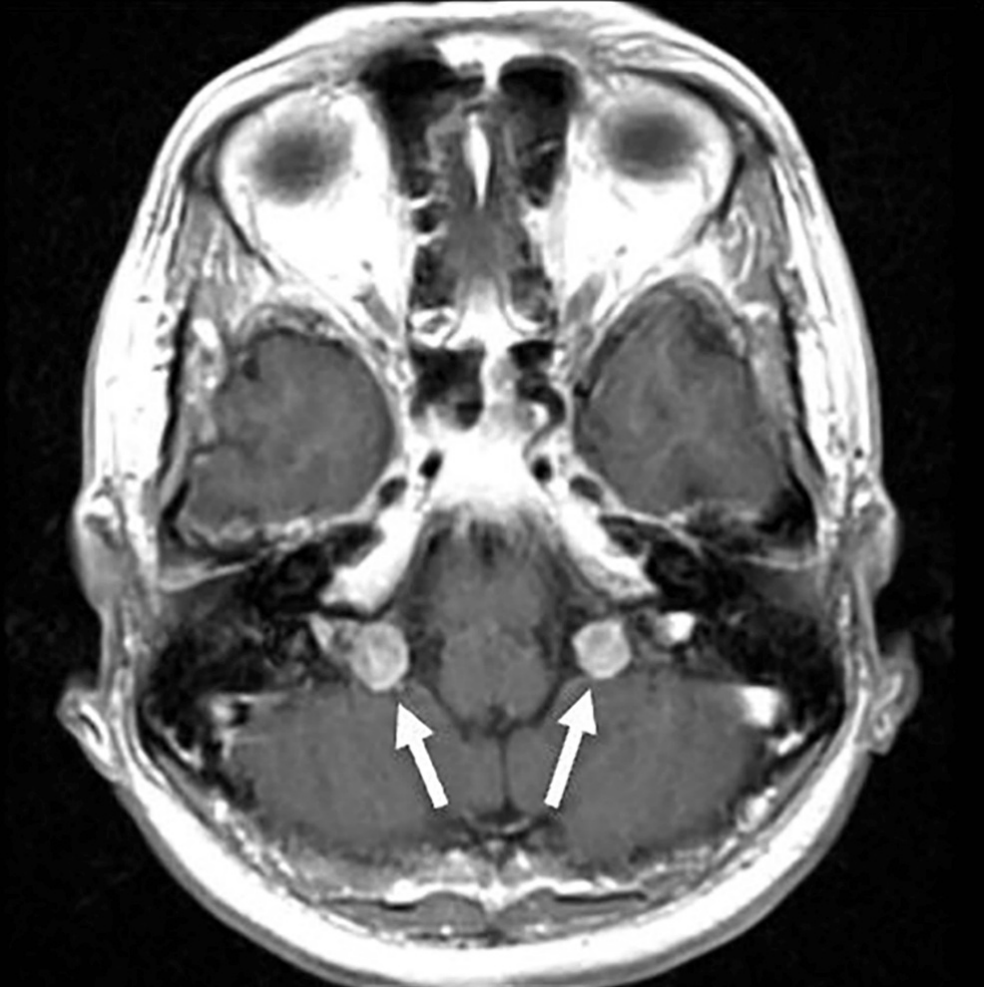
T1 MRI post-contrast demonstrating bilateral round enhancing cerebellopontine angle jugular foramen lesions (arrows), 1.1 cm on the right and 0.9 cm on the left in maximal dimension.

The patient was a 58-year-old female who first presented to a clinic in March 2019 with a complaint of hoarseness. The hoarseness began in 2015 and was initially thought to be related to her gastroesophageal reflux disease. Imaging obtained by her laryngologist showed bilateral jugular foramen tumors and thoracic lymphadenopathy (Figure 1), which led to the patient being referred to an otology/neurotology specialist. The patient underwent a neck dissection seven months later to expose the vagus nerve and isolate the great vessels in preparation for a transmastoid approach to the skull base. Intraoperatively, an extreme enlargement and thickening of the vagus nerve were found, which appeared consistent with a vagal schwannoma, and a separate mass of the cervical sympathetic trunk located superior to this in the parapharyngeal space. Biopsies from the vagus nerve were sent for frozen pathology, which was suspicious for schwannoma. The patient was discharged on postoperative day one and reported no change in her voice or swallowing at her first post-operative visit.

**Figure 2:**
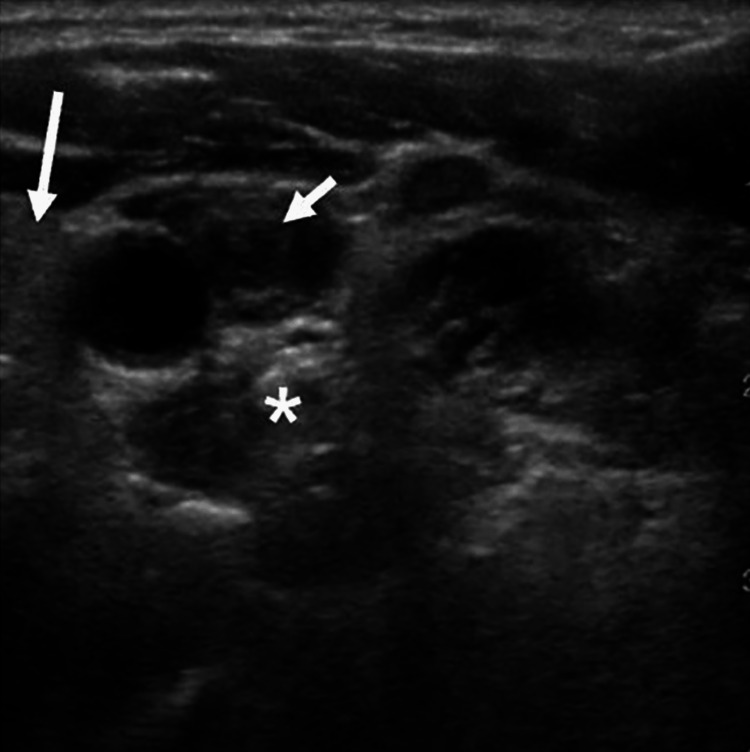
Preoperative ultrasound of the left neck demonstrating significant thickening of the vagus nerve (short arrow) and cervical sympathetic plexus (asterisk). Left thyroid (long arrow).

The patient was examined and an ultrasound of the neck was performed, which was performed to get a detailed view of the thyroid gland and cervical lymph nodes. The technique used for the ultrasound examination involved the use of a portable ultrasound machine and Doppler gel, and the examination included the thyroid gland, trachea, and bilateral lymph node levels I-VI. The findings of the ultrasound revealed that the left and right thyroid lobes were both normal in appearance and without any visualized nodules, cysts, or other irregularities. In the right neck, the carotid artery and internal jugular vein were patent, and the submandibular gland was normal in appearance. However, at the level of the superior pole of the thyroid, there was a heterogenous mass effect that was immediately adjacent to the carotid artery and appeared to be within the carotid sheath itself (Figure 2). Similarly, in the left lateral neck, the carotid artery and internal jugular vein were patent, and the submandibular gland was normal in appearance. However, there was also a heterogenous mass effect at the level of the superior pole of the thyroid that was immediately adjacent to the carotid artery and appeared to be within the carotid sheath itself. On the left side, this mass extended more superiorly, and on longitudinal view, it had a tubular shape, possibly consistent with vagal schwannoma.

The patient tolerated the procedure well, and there were no complications. However, given the patient's history of bilateral vocal cord paralysis and the presence of bilateral cerebellopontine angle/jugular foramen masses as well as pleural lymphadenopathy of uncertain origin, the doctor referred the patient to a colleague in neuroradiology for an ultrasound-guided biopsy of the abnormal neck masses to help establish a pathologic diagnosis.

The CT scan of the patient's neck with contrast revealed that the patient has heterogenous left and right parotid glands, symmetrically prominent right and left submandibular glands, an unremarkable thyroid gland, symmetrically prominent pharyngeal tonsils, a patent airway, symmetric laryngeal ventricles, a right dorsal 5 mm tracheal diverticulum just below the origin of the cervical esophagus, left level IV lymphadenopathy extending up from the chest, bilateral posteriorly projecting aneurysms or infundibula at the expected origins of the posterior communicating arteries of the right and left internal carotid arteries measuring 3 mm on the right and 2-3 mm on the left, unremarkable visualized osseous structures, well-aerated paranasal sinuses and mastoid air cells, and a chronic defect in the left medial orbital wall.

The MRI report of the brain showed that the corpus callosum, pituitary gland, and cerebellar tonsils are normal. There was no abnormal enhancement and no sign of infarct or hemorrhage, but there were bilateral rounded enhancing cerebellopontine angle mass lesions measuring up to 1.1 cm on the right and up to 0.9 cm on the left. These were T1 and T2 hypointense and hypointense on CT. There were also non-enhancing scattered foci of abnormal T2 fluid-attenuated inversion recovery (FLAIR) signal hyperintensity in the bilateral deep cerebral white matter, which is most likely an atypical manifestation of mild chronic small vessel white matter ischemic disease. The meninges and extra-axial spaces were normal, major and arterial venous flow voids were normal, and the sinuses and mastoids were normal, but there was a chronic defect in the medial left orbital wall.

The operative report describes a surgical procedure performed on a patient to investigate and sample a suspected vagal schwannoma, a benign tumor that develops on the vagus nerve. The procedure was performed under general endotracheal anesthesia and the patient was placed in a supine position. The surgical team used a hockey stick incision to gain access to the neck area, and nerve monitoring electrodes were placed in the palate, trapezius, and face for neuromonitoring. The surgical team then dissected the various layers of tissue in the neck, carefully identifying and preserving the external and internal carotid artery, the internal jugular vein, the hypoglossal nerve, and the spinal accessory nerve.

**Figure 3:**
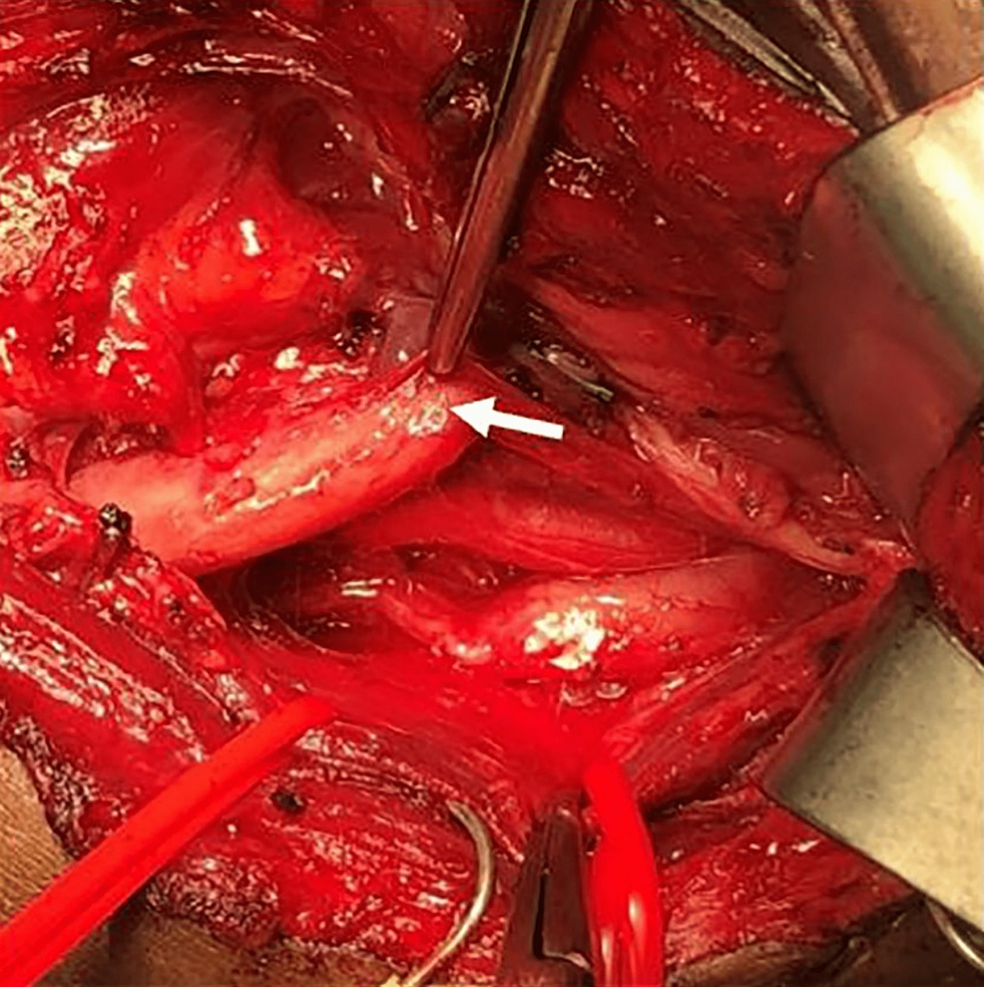
Intraoperative photograph demonstrating thickened vagus nerve that was biopsied (arrow).

As the surgical team entered the carotid sheath and began to trace the vagus nerve proximally, they observed that the nerve appeared normal in appearance above the level of the carotid bifurcation, but that it was enlarged and fibrous in appearance below the bifurcation, consistent with a schwannoma (Figure 3). The nerve did not stimulate when stimulated by the surgical team. Additionally, the team identified a separate schwannoma of the cervical sympathetic trunk located superior to the vagus schwannoma in the parapharyngeal space.

Given the presence of multifocal tumors in multiple nerves and the bilateral nature of the patient's disease, the decision was made to not attempt a complete resection of the schwannoma at this time. Instead, the surgical team took samples of the vagus nerve tumor for pathology and sent them for frozen pathology examination. The frozen pathology confirmed the presence of a schwannoma.

The surgical team then closed the neck and the patient was transferred to the post-anesthesia care unit in stable condition with no complications reported. The patient was extubated to room air and the postoperative plan was to keep the patient stable in the post-anesthesia care unit.

The pathology report described the results of a biopsy and resection of a lymph node and nerve in the left neck area. The lymph node was described as enlarged and having mild reactive changes. The nerve was described as having granulomatous inflammation, which is characterized by the presence of granulomas, which are clusters of inflammatory cells. The report notes that special stains have been ordered to investigate the possibility of an infectious etiology, but the results are not yet available.

**Figure 4:**
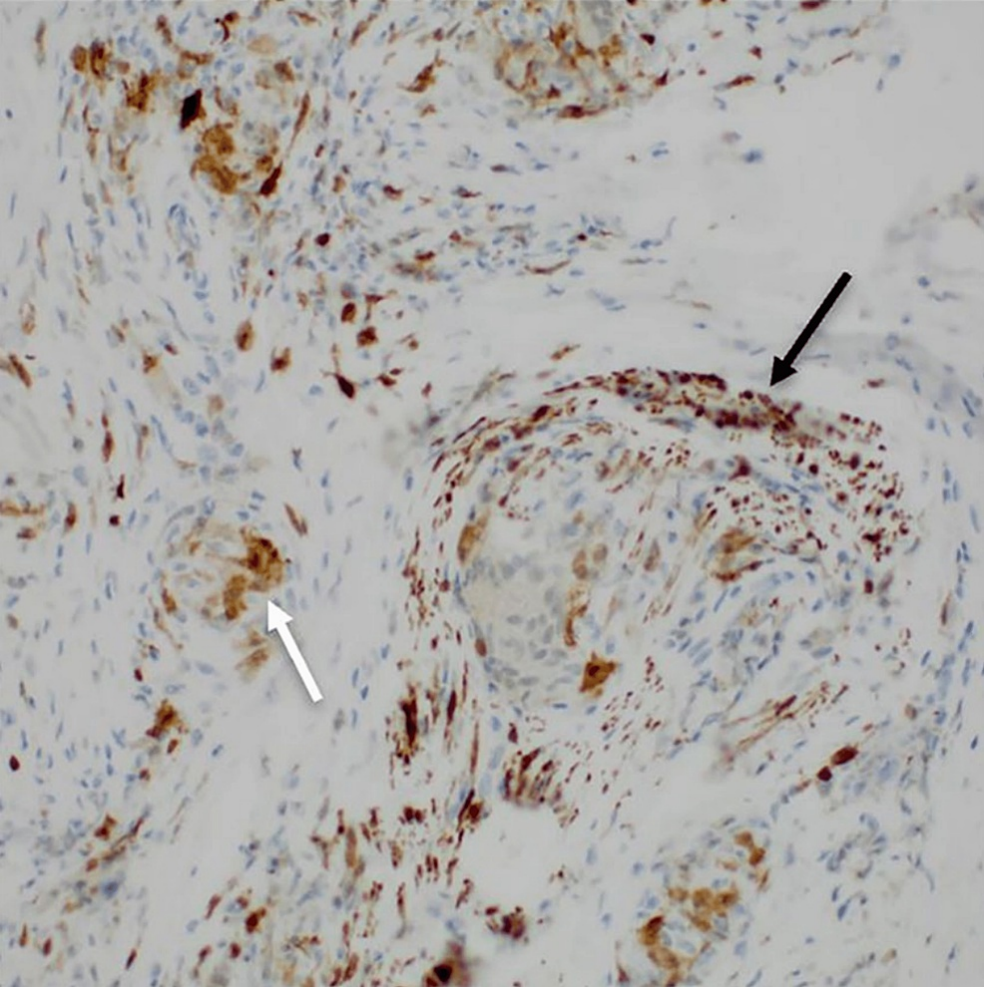
S100 stain demonstrates bundles of nerve twigs running through dense fibrous tissue and highlights giant cells (arrows).

The addendum to the report states that special stains and immunohistochemistry were performed on the nerve tissue and that these tests did not reveal any discernible organisms. However, the S100 immunohistochemistry did highlight bundles of nerve twigs running through the dense fibrous tissue and the giant cells (Figure 4). Based on these findings, and the fact that the patient had similar findings in the lung, the diagnosis of chronic sarcoidosis was considered most likely. Neurosarcoidosis, which is sarcoidosis affecting the cranial nerves, has been reported in the medical literature [2].

## Discussion

This case of BVFP caused by neurosarcoidosis is novel in that it is the first case of its kind. The patient, a 58-year-old female, initially presented with hoarseness, which was initially thought to be related to her gastroesophageal reflux disease. Imaging showed bilateral jugular foramen tumors and thoracic lymphadenopathy, leading to a referral to an otology/neurotology specialist. The patient underwent a neck dissection, which revealed an extreme enlargement and thickening of the vagus nerve and a separate mass of the cervical sympathetic trunk. Biopsies were sent for frozen pathology, which was suspicious for a schwannoma. The patient was referred to a colleague in neuroradiology for an ultrasound-guided or CT-guided biopsy of the abnormal neck masses to establish a pathologic diagnosis. CT scan revealed heterogenous left and right parotid glands, symmetrically prominent right and left submandibular glands, an unremarkable thyroid gland, symmetrically prominent pharyngeal tonsils, and a patent airway. MRI of the brain showed bilateral rounded enhancing cerebellopontine angle mass lesions and non-enhancing scattered foci of abnormal T2-FLAIR signal hyperintensity in the bilateral deep cerebral white matter.

Neurosarcoidosis is a rare complication of sarcoidosis, a chronic inflammatory disease of unknown cause that can affect multiple organs. In neurosarcoidosis, sarcoid granulomas (clumps of inflammatory cells) form in the nervous system, typically in the brain and spinal cord. The exact mechanisms of neurosarcoidosis are not well understood, but it is believed that the granulomas cause inflammation and damage to the affected nerves and tissues [1,2,4].

This case is unique in its findings in that it is a patient with an unusually high number of neurological symptoms, including multiple cranial nerve involvement, seizures, and cognitive impairment. This combination of symptoms is uncommon and highlights the potential for sarcoidosis to have a severe impact on the nervous system [3].

**Figure 5:**
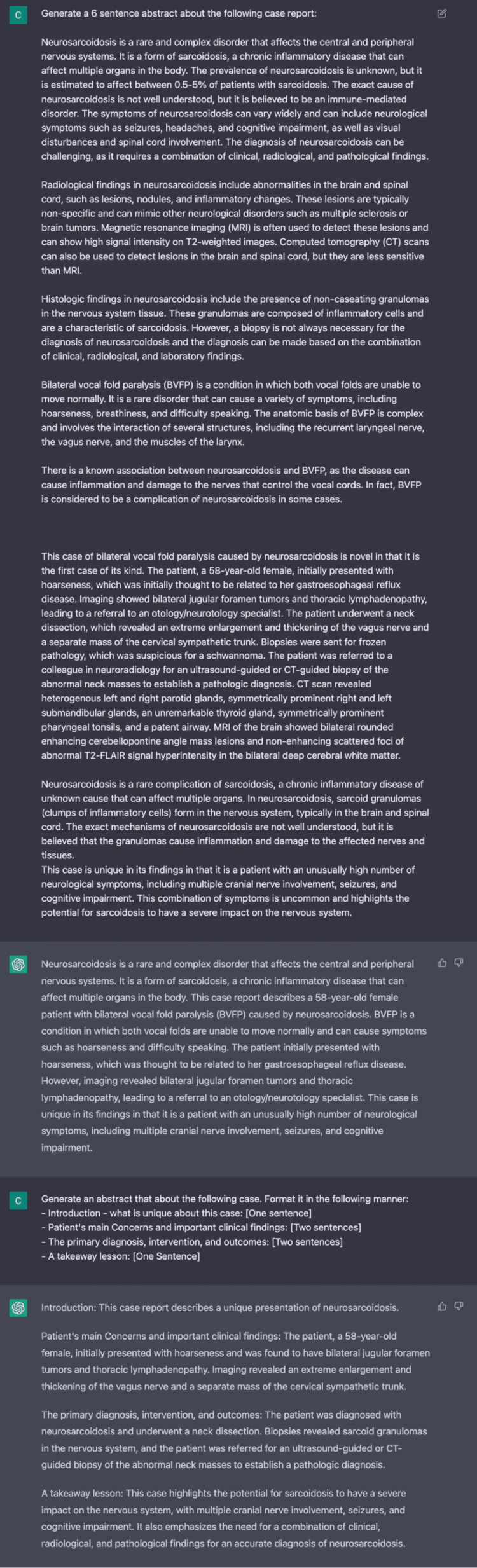
This figure shows ChatGPT reprocessing the body of the case report to generate an abstract. It is able to reprocess its response using a semi-persistent conversation thus allowing fine-tuning of the content and format of its responses.

**Figure 6:**
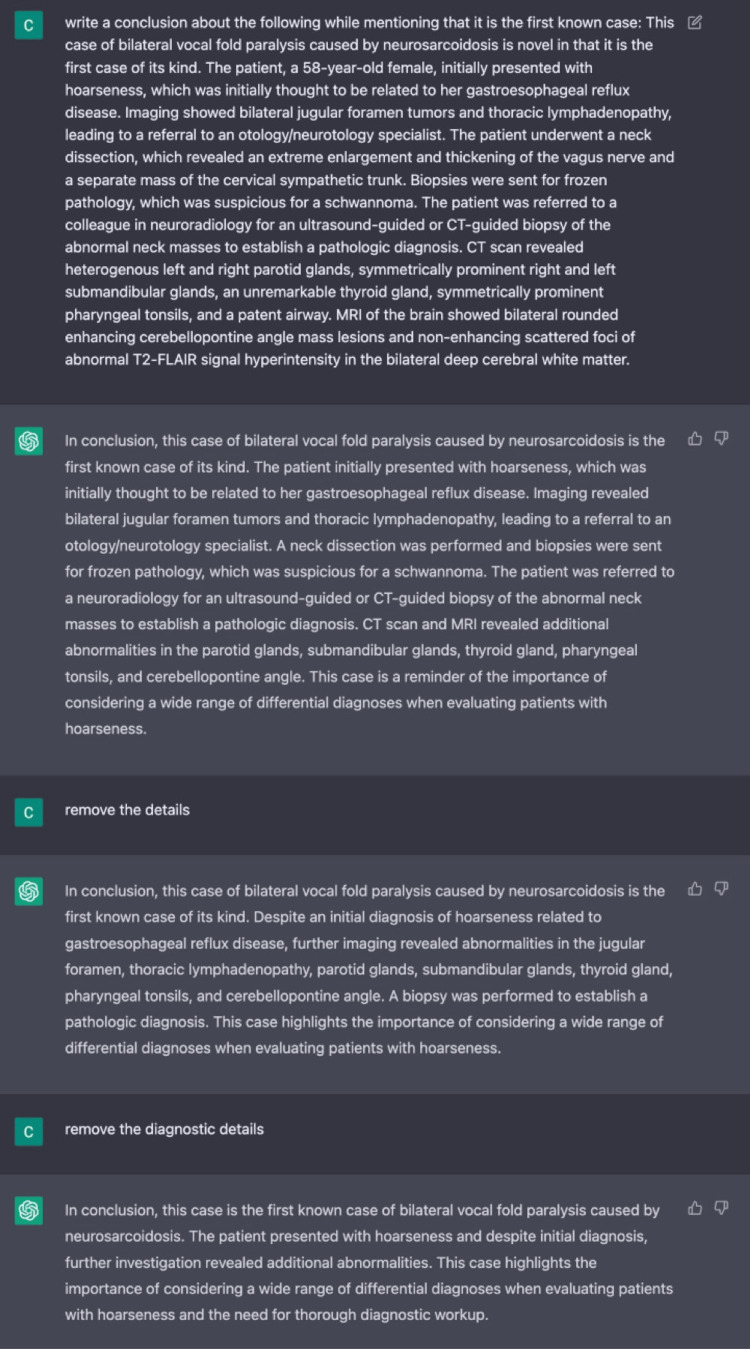
This figure shows ChatGPT reprocessing the body of the case report to generate a conclusion. Using the aforementioned semi-persistent conversation, it can be prompted to add or remove information.

Lastly, as this report is a fully ChatGPT/NLP-generated case report, it can be compared against the original article, which serves as the benchmark for case report quality [5]. Various prompts used in the generation of this report can be seen in Figures 5, 6.

## Conclusions

In conclusion, this case is the first known case of BVFP caused by neurosarcoidosis. The patient presented with hoarseness and despite the initial diagnosis, further investigation revealed additional abnormalities. This case highlights the importance of considering a wide range of differential diagnoses when evaluating patients with hoarseness and the need for a thorough diagnostic workup.
